# A standard operating procedure for King’s ALS clinical staging

**DOI:** 10.1080/21678421.2018.1556696

**Published:** 2019-02-18

**Authors:** Rubika Balendra, Ahmad Al Khleifat, Ton Fang, Ammar Al-Chalabi

**Affiliations:** 1 Department of Neurodegenerative Disease, UCL Institute of Neurology, London, UK,; 2 Department of Genetics, Evolution and Environment, Institute of Healthy Ageing, University College London, London, UK, and; 3 Department of Basic and Clinical Neuroscience, Maurice Wohl Clinical Neuroscience Institute, King’s College London, London, UK

**Keywords:** Clinical trials, survival, prognostic, staging,&nbsp, standard operating procedure

## Abstract

*Objective*: Clinical stages in amyotrophic lateral sclerosis (ALS) can be measured using a simple system based on the number of CNS regions involved and requirement for gastrostomy or noninvasive ventilation (NIV). We aimed to design a standard operating procedure (SOP) to define the standardized use and application of the King’s staging system. *Methods*: We designed a SOP for the King’s staging system. We wrote case vignettes representative of ALS patients at different disease stages. During two workshops, we taught health care professionals how to use the SOP, then asked them to stage the vignettes using the SOP. We measured the extent to which SOP staging corresponded with correct clinical stage. *Results*: The reliability of staging using the SOP was excellent, with a Spearman’s Rank coefficient of 0.95 (*p* < 0.001), and was high for different groups of health care professionals, and for those with different levels of experience in ALS. The limits of agreement between SOP staging and actual clinical stage lie within a single stage, confirming that there is a clinically acceptable level of agreement between staging using the SOP and actual King’s clinical stage. There were also no systematic biases of the SOP over the range of stages, either for over-staging or under-staging. *Conclusions*: We have demonstrated that the staging SOP provides a reliable method of calculating clinical stages in ALS patients and can be used prospectively by a range of health care professionals with different levels of experience, as for example may be the case in multicentre clinical trials.

## Introduction

Amyotrophic lateral sclerosis (ALS) is progressive neurodegenerative disease affecting upper and lower motor neurons. Two recent staging systems have been proposed to measure the clinical progression of disease (1,2). The King’s staging system consists of five disease stages, with Stage 5 being death. Stages 1–3 are based upon the number of El Escorial central nervous system (CNS) regions involved in the disease, measured by weakness, wasting, spasticity, dysphagia, or dysarthria. Stage 4 is nutritional failure, defined by the requirement for gastrostomy, or respiratory failure, defined by the requirement for noninvasive ventilation (NIV), and based upon the National Institute for Clinical Excellence MND Guidelines (Supplemental Material). The Milano–Torino (MiToS) Staging system comprises six stages, based on functional impairment as assessed by the revised ALS Functional Rating Scale (ALSFRS-R) (2). These staging systems are complementary, with the King’s clinical staging system closely linked to anatomical spread, and the MiToS system closely tracking functional spread. As a result, King’s clinical staging has a higher resolution in early-mid diseases stages and the MiToS system in late disease stages (3). In the King’s staging system, there is more homogeneity between patients in the same stage, and a greater discrimination between patients in different disease stages (4).

In ALS, these staging systems correlate with a decline in functional measures, health utility and quality of life scores, and an increase in socioeconomic costs, comprising costs of healthcare, and loss of productivity (2,5–7). ALS stages have validity as meaningful outcome measures in ALS clinical trials, as they help to account for the inherent heterogeneity in patient populations, and can facilitate the development of drugs which differing efficacies throughout disease progression (8,9). Progression to a higher ALS disease stage is now being utilized as a primary outcome measure in Phase II randomized controlled trial (10) and has been used to determine the stage at which Riluzole prolongs survival in ALS in a retrospective study (11). Staging can be used to select patients for clinical studies, for example investigating neuroimaging biomarkers in early disease stages (12). Furthermore, staging has utility in mapping to neuroimaging and biochemical biomarkers in ALS patients, correlating with reduction in white matter integrity in ALS patients with repeat expansions in the *C9orf72* gene on neuroimaging (13), and with higher levels of CSF neurofilament light chain (14,15). Staging correlates with other important disease parameters, including sustained and forced vital capacity (7), and energy expenditure (16). Mitochondrial dysfunction is involved in the pathogenesis of ALS (17), and mitochondrial activity detected in patient peripheral blood mononuclear cells decreases with increasing disease stage (18), indicating that staging may correlate with underlying etiopathogenic mechanistic markers.

ALS is on a clinical, genetic and pathological spectrum with frontotemporal dementia (FTD). Up to 15% of people with ALS have a diagnosis of FTD, and cognitive impairment occurs in about 50% of people with ALS (19,20). Measures of behavior and cognition are reduced in later ALS disease stages (21–23). Moreover, specific staging systems to measure the extent of cognitive involvement in ALS have been developed (24,25) and could be used in parallel with ALS disease staging systems.

We have previously shown that King’s clinical stages can be reliably estimated retrospectively in preexisting datasets from the ALSFRS-R (26). In order for the King’s staging system to be applied by different health care professionals of varying levels of experience working in ALS, as for example may be the case in a multicentre clinical trial, we designed a standard operating procedure (SOP) for the use of the King’s system. We then investigated whether it could be used by a variety of health care professionals.

## Materials and methods

We wrote a SOP for using the King’s clinical staging system for ALS (1) (Supplementary Material). We created 17 case vignettes of patients with ALS, representing a spectrum of cases with different stages of disease, ranging from Stage 1–4 (Supplementary Material). In 2016 and 2017, we ran two staging workshops during the European Network for the Cure of ALS (ENCALS) meetings. Participants included doctors, nurses, allied health care professionals, and researchers with varying lengths of experience in ALS. We collected data on each participant’s occupation and length of time working in ALS. During each workshop, we provided training in how to apply the staging SOP, and asked participants to stage the vignettes using the SOP. In the first workshop, all 17 vignettes were included in the study, and in the second workshop 10 of the vignettes were included.

### Statistical analysis

To measure the reliability of staging using the SOP across the entire cohort, we calculated a Spearman’s rank correlation coefficient between the actual King’s clinical stage and the King’s stage assessed according to the SOP. We also calculated Spearman’s rank correlation coefficients for different health care professional groups included in the study (doctors, nurses, and allied health care professionals), and for those with less than 10 years, or 10 years or greater experience working in ALS.

As a further step to investigate the reliability of using the SOP to calculate clinical stage, we used the Bland–Altman method to calculate the difference between actual King’s clinical stage and King’s stage calculated by participants using the SOP, and the mean of the correct clinical stage and stage using the SOP, determining the limits of agreement between these (27,28). To test for any systematic bias in the SOP, leading to over-estimation or under-estimation of stage, we calculated the Spearman’s rank correlation coefficient between the difference between actual King’s clinical stage and stage using the SOP, and also the mean of the actual King’s clinical stage and stage using the SOP (28).

Analyses were performed in SPSS version 20.0 (SPSS Inc., Chicago, IL) and GraphPad Prism version 6.07 (GraphPad Software, La Jolla, CA).

## Results

**Figure 1:**
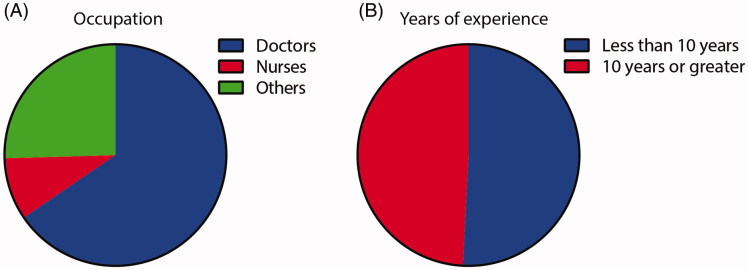
Demographics of participants included in the study. (A) The study consisted of 61 participants in total, with doctors (65.5%), nurses (9.1%), and allied health care professionals (25.5%) represented in the cohort. (B) There was an even distribution between those with less than 10 years’ experience working in ALS (50.8%) and those with 10 years’ or greater experience (48.2%).

The study consisted of 61 participants in total, with doctors (65.5%), nurses (9.1%), and other allied health care professionals (25.5%) represented in the cohort (Figure 1(A)). There was an even distribution between those with less than 10 years’ experience working in ALS (50.8%) and those with 10 years’ or greater experience (48.2%) (Figure 1(B)).

### Use of a standard operating procedure leads to reliable clinical staging

**Figure 2:**
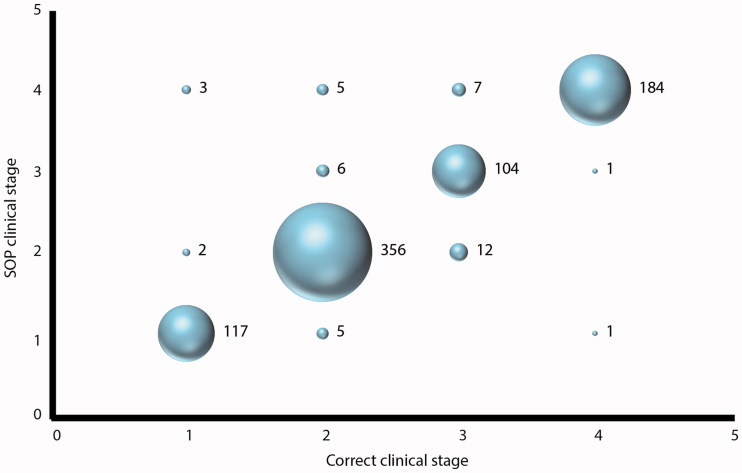
Use of a standard operating procedure leads to reliable clinical staging. Participants were trained in how to use the staging standard operating procedure (SOP, Supplementary Material), and most correctly staged case vignettes using the SOP. The numbers to the right of each bubble represent the number of answers within each group.

Across the cohort, we found that use of a SOP led to a high reliability of clinical staging of vignettes. The correlation between staging of the clinical vignettes using the SOP and the actual King’s clinical stage was Spearman’s rho = 0.95, *p* < 0.001 (Figure 2). There was a very strong correlation between staging using the SOP and the actual King’s clinical stage for every health care professional group, with similar correlations between each group: doctors (Spearman’s rho = 0.95, *p* < 0.001), nurses (Spearman’s rho = 0.93, *p* < 0.001), and allied health care professionals (Spearman’s rho = 0.94, *p* < 0.001). The correlation between staging using the SOP and the actual King’s clinical stage was the same for those with at least 10 years’ experience working with patients with ALS (Spearman’s rho = 0.95, *p* < 0.001) as for those with less than 10 years’ experience (Spearman’s rho = 0.95, *p* < 0.001). Overall, most participants correctly staged case vignettes in Stages 1 (95.9%), 2 (95.7%), 3 (84.6%), and 4 (98.9%) using the SOP (Figure 2).

We used the Bland–Altman method to calculate the difference between correct clinical stage and stage using the SOP for all pairs and the mean of the stages for all pairs. The mean of the difference in stage between the two methods was 0.01 (95% CI of the mean −0.01–0.04, standard deviation 0.35) with the 95% confidence limits of agreement lying between −0.69 and 0.71. Therefore, the limits of agreement lie within a single stage, confirming that there is a clinically acceptable level of agreement between staging using the SOP and actual King’s clinical stage. To test for systematic bias of the SOP over the range of stages, leading to over-staging or under-staging, we calculated a Spearman’s rank correlation coefficient between the differences in stages and the means of stages, which showed a negligible relationship between the two (Spearman’s rho = 0.069, *p* = 0.05). Therefore, there are unlikely to be any systematic biases in staging when using the SOP. As a further confirmation of this, there was a similar number of cases where staging using the SOP led to a higher stage than the actual clinical stage (23 cases) and cases where SOP staging led to a lower stage (20 cases), and these erroneously staged cases amounted to only 5.35% in total of the whole study.

### Use of the standard operating procedure for patients with stage 4 disease

**Figure 3:**
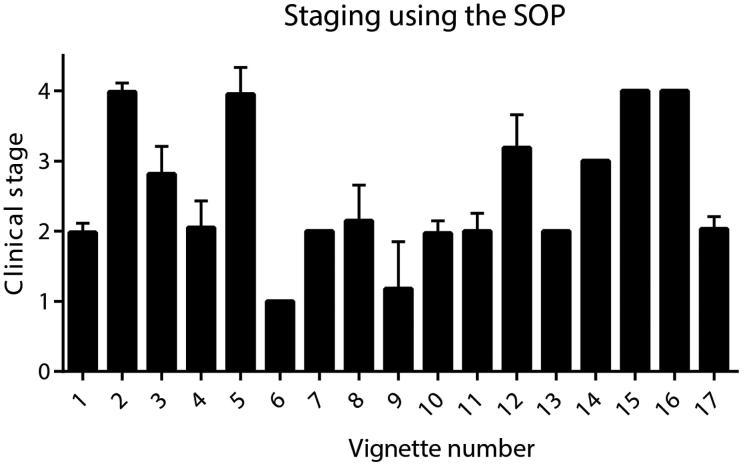
Variability of scores for staging using the SOP for each case vignette. Bars represent the mean and standard deviation of scores for staging using the SOP for each vignette. Variability in the answers was greatest for vignettes 8, 9, and 12.

Variability in the answers for staging using the SOP was greatest for vignettes 8, 9, and 12 (Figure 3). In vignette 9, a gastrostomy had been inserted for a reason other than ALS, due to oropharyngeal malignancy. Some participants had staged this case as Stage 4, despite the gastrostomy not being required as an intervention for ALS-related dysphagia. The SOP clarifies that when the gastrostomy is required as an intervention for ALS-related dysphagia, Stage 4 is reached and has clear parameters for when these criteria are fulfilled. In vignettes 8 and 12, the patients did not yet meet the criteria for respiratory failure; however, some participants had staged these cases as Stage 4. The SOP clarifies that Stage 4 is only reached when the UK National Institute of Health and Care Excellence MND guidelines for respiratory failure are reached, and the guidelines are stated in a summarized format within the SOP, although relaxed, country-specific guidelines are also acceptable. Furthermore, the average staging for vignette 3 across the participants was 2.8, demonstrating that it was relatively under-staged, as the correct answer for this vignette was Stage 3. This is likely due to some participants not classifying a brisk jaw jerk on examination, without evidence of dysarthria or dysphagia, as indicative of brainstem involvement. However, we have clarified in the SOP that this sign does indicate brainstem disease.

## Discussion

We have shown that using an SOP provides a highly reliable method of calculating clinical stages in patients with ALS. The 95% confidence limits of agreement of staging using the SOP were within less than a clinical stage and there were no clear systematic biases leading to over- or under-staging, further confirming that using the SOP for clinical staging is reliable.

We have demonstrated that using an SOP is effective across different groups of health care professionals and across different levels of experience working within ALS. ALS staging systems have utility in stratifying patients in clinical studies, as a marker of disease progression when assessing validity of biomarkers, and as outcome measures in clinical trials. For their use to be effective, it is critical the system is simple to understand and apply. This is of clear importance in ensuring staging can be applied reliably by different individuals, separated in space and time, as is the case, for example in multicentre clinical trials. Participants were attendees at an ALS specialist meeting, therefore, are likely to be representative of the individuals that would use the SOP and staging system in the future in clinical studies and clinical trials. The impact of this study is that the SOP can now be implemented widely in future studies where it would be useful to prospectively collect staging data.

We found that the vignettes that were most variable were sometimes over-estimated as Stage 4. We have clarified in the SOP the exact definitions of when Stage 4 is reached, either when gastrostomy or NIV are required. It is likely that as this SOP is used more frequently alongside further training sessions, its repeated use will improve the reliability of clinical staging further. Some flexibility in the definitions of Stage 4 may, however, be necessary for implementations in different health systems.

Although the agreement between staging using the SOP and actual King’s clinical stage was high, it was not perfect. However, we found that the limits of agreement lay within a single stage, with systematic biases being negligible, and we detected a very low rate of errors (∼5%) represented equally by cases of over- and under-staging, indicating that the extent of agreement is clinically acceptable. Overall, this suggests that the SOP is straightforward to understand as written.

A potential limitation of this study is that staging using the SOP was performed directly after a training session was delivered, and the accuracy of staging may have, therefore, been at its highest at this time. However, we would expect these reliability measures to continue to improve after repeated use of the SOP on subsequent occasions, and the SOP is now readily available for users to refer to in the future as required. A further limitation is that this cohort represents a relatively small number of participants with an overrepresentation of doctors compared to nurses and other allied health care professionals. Further studies are, therefore, required to validate whether the SOP is reliable across a larger and more diverse group of health care professionals. Future validation could be achieved using an online survey platform.

In conclusion, here we have presented a SOP for the application of the King’s clinical staging system for ALS. This SOP makes staging reliable and simple to apply to a variety of representative patient cases, and by a range of health care professionals with different levels of experience in ALS.

## Supplementary Material

Supplementary_Material_Revised.docx

## References

[1] Roche JC, Rojas-Garcia R, Scott KM, Scotton W, Ellis CE, Burman R, et al. A proposed staging system for amyotrophic lateral sclerosis. Brain J Neurol. 2012;135:847–52. 10.1093/brain/awr351 PMC3286327 22271664

[2] Chio A, Hammond ER, Mora G, Bonito V, Filippini G Development and evaluation of a clinical staging system for amyotrophic lateral sclerosis. J Neurol Neurosurg Psychiatry. 2015;86:38–44. 24336810 10.1136/jnnp-2013-306589

[3] Fang T, Al Khleifat A, Stahl DR, Lazo La Torre C, Murphy C, UK-MND LiCALS, et al. Comparison of the King’s and MiToS staging systems for ALS. Amyotroph Lateral Scler Frontotemporal Degener. 2017;18:227–32. 28054828 10.1080/21678421.2016.1265565 PMC5425622

[4] Ferraro D, Consonni D, Fini N, Fasano A, Del Giovane C, Emilia Romagna Registry for ALSG, et al. Amyotrophic lateral sclerosis: a comparison of two staging systems in a population-based study. Eur J Neurol. 2016;23:1426–32. 27238551 10.1111/ene.13053

[5] Jones AR, Jivraj N, Balendra R, Murphy C, Kelly J, Thornhill M, et al. Health utility decreases with increasing clinical stage in amyotrophic lateral sclerosis. Amyotroph Lateral Scler Frontotemporal Degener. 2014;15:285–91. 24641613 10.3109/21678421.2013.872149

[6] Oh J, An JW, Oh SI, Oh KW, Kim JA, Lee JS, et al. Socioeconomic costs of amyotrophic lateral sclerosis according to staging system. Amyotroph Lateral Scler Frontotemporal Degener. 2015;16:202–8. 25646865 10.3109/21678421.2014.999791

[7] Pinto S, de Carvalho M Comparison of slow and forced vital capacities on ability to predict survival in ALS. Amyotroph Lateral Scler Frontotemporal Degener. 2017;18:528–33. 28741375 10.1080/21678421.2017.1354995

[8] Balendra R, Jones A, Jivraj N, Steen IN, Young CA, Shaw PJ, et al. Use of clinical staging in amyotrophic lateral sclerosis for phase 3 clinical trials. J Neurol Neurosurg Psychiatry. 2015;86:45–9. 24463480 10.1136/jnnp-2013-306865

[9] Tramacere I, Dalla Bella E, Chio A, Mora G, Filippini G, Lauria G, et al. The MITOS system predicts long-term survival in amyotrophic lateral sclerosis. J Neurol Neurosurg Psychiatry. 2015;86:1118–5. 10.1136/jnnp-2014-310176 25886781

[10] Bella ED, Tramacere I, Antonini G, Borghero G, Capasso M, Caponnetto C, et al. Protein misfolding, amyotrophic lateral sclerosis and guanabenz: protocol for a phase II RCT with futility design (ProMISe trial). BMJ Open. 2017;7:e015434. 10.1136/bmjopen-2016-015434 PMC5724081 28801400

[11] Fang T, Al Khleifat A, Meurgey JH, Jones A, Leigh PN, Bensimon G, et al. Stage at which riluzole treatment prolongs survival in patients with amyotrophic lateral sclerosis: a retrospective analysis of data from a dose-ranging study. Lancet Neurol. 2018;17:416–22. 29525492 10.1016/S1474-4422(18)30054-1 PMC5899963

[12] Trojsi F, Caiazzo G, Di Nardo F, Fratello M, Santangelo G, Siciliano M, et al. High angular resolution diffusion imaging abnormalities in the early stages of amyotrophic lateral sclerosis. J Neurol Sci. 2017;380:215–22. 28870572 10.1016/j.jns.2017.07.039

[13] Floeter MK, Danielian LE, Braun LE, Wu T Longitudinal diffusion imaging across the C9orf72 clinical spectrum. J Neurol Neurosurg Psychiatry. 2018;89:53–60. 29054917 10.1136/jnnp-2017-316799 PMC6454927

[14] Gaiani A, Martinelli I, Bello L, Querin G, Puthenparampil M, Ruggero S, et al. Diagnostic and prognostic biomarkers in amyotrophic lateral sclerosis: neurofilament light chain levels in definite subtypes of disease. JAMA Neurol. 2017;74:525–32. 28264096 10.1001/jamaneurol.2016.5398 PMC5822207

[15] Puentes F, Topping J, Kuhle J, van der Star BJ, Douiri A, Giovannoni G, et al. Immune reactivity to neurofilament proteins in the clinical staging of amyotrophic lateral sclerosis. J Neurol Neurosurg Psychiatry. 2014;85:274–8. 24078718 10.1136/jnnp-2013-305494

[16] Lee J, Baek H, Kim SH, Park Y Association between estimated total daily energy expenditure and stage of amyotrophic lateral sclerosis. Nutrition. 2017;33:181–6. 27544003 10.1016/j.nut.2016.06.007

[17] Hardiman O, Al-Chalabi A, Chio A, Corr EM, Logroscino G, Robberecht W, et al. Amyotrophic lateral sclerosis. Nat Rev Dis Primers. 2017;3:17071. 28980624 10.1038/nrdp.2017.71

[18] Ehinger JK, Morota S, Hansson MJ, Paul G, Elmer E Mitochondrial dysfunction in blood cells from amyotrophic lateral sclerosis patients. J Neurol. 2015;262:1493–503. 25893255 10.1007/s00415-015-7737-0

[19] Ringholz GM, Appel SH, Bradshaw M, Cooke NA, Mosnik DM, Schulz PE Prevalence and patterns of cognitive impairment in sporadic ALS. Neurology. 2005;65:586–90. 16116120 10.1212/01.wnl.0000172911.39167.b6

[20] Ling SC, Polymenidou M, Cleveland DW Converging mechanisms in ALS and FTD: disrupted RNA and protein homeostasis. Neuron. 2013;79:416–38. 23931993 10.1016/j.neuron.2013.07.033 PMC4411085

[21] Burke T, Pinto-Grau M, Lonergan K, Bede P, O’Sullivan M, Heverin M, et al. A cross-sectional population-based investigation into behavioral change in amyotrophic lateral sclerosis: subphenotypes, staging, cognitive predictors, and survival. Ann Clin Transl Neurol. 2017;4:305–17. 28491898 10.1002/acn3.407 PMC5420811

[22] Trojsi F, Santangelo G, Caiazzo G, Siciliano M, Ferrantino T, Piccirillo G, et al. Neuropsychological assessment in different King’s clinical stages of amyotrophic lateral sclerosis. Amyotroph Lateral Scler Frontotemporal Degener. 2016;17:228–35. 26905940 10.3109/21678421.2016.1143513

[23] Christopher JC, Lonergan K, Chiwera T, Booth T, Chandran S, Colville S, et al. ALS specific cognitive and behaviour changes associated with advancing disease stage in ALS. Neurology. 2018;91:e1370–e80. 30209236 10.1212/WNL.0000000000006317 PMC6177274

[24] Lule D, Bohm S, Muller HP, Aho-Ozhan H, Keller J, Gorges M, et al. Cognitive phenotypes of sequential staging in amyotrophic lateral sclerosis. Cortex. 2018;101:163–71. 29477906 10.1016/j.cortex.2018.01.004

[25] Abrahams S, Newton J, Niven E, Foley J, Bak TH Screening for cognition and behaviour changes in ALS. Amyotroph Lateral Scler Frontotemporal Degener. 2014;15:9–14. 23781974 10.3109/21678421.2013.805784

[26] Balendra R, Jones A, Jivraj N, Knights C, Ellis CM, Burman R, et al. Estimating clinical stage of amyotrophic lateral sclerosis from the ALS functional rating scale. Amyotroph Lateral Scler Frontotemporal Degener. 2014;15:279–84. 24720420 10.3109/21678421.2014.897357

[27] Bland JM, Altman DG Statistical methods for assessing agreement between two methods of clinical measurement. Lancet. 1986;1:307–10. 2868172

[28] Sedgwick P Limits of agreement (Bland-Altman method). BMJ. 2013;346:f1630. 23502707 10.1136/bmj.f1630

