# Supplementary material for: A standard operating procedure for King’s ALS clinical staging
Source: Amyotroph Lateral Scler Frontotemporal Degener. 2019 Feb 18;20(3-4):159–64. doi: 10.1080/21678421.2018.1556696 (PMC6558284; doi:10.1080/21678421.2018.1556696)
Supplement: Supplementary_Material_Revised.docx [file IAFD_A_1556696_SM3064.docx]

**Supplementary Material**

**A standard operating procedure for King’s ALS clinical staging**

Rubika Balendra^1,2,*^, Ahmad Al Khleifat^3,*^, Ton Fang^3^, Ammar Al-Chalabi^3^

1. Department of Neurodegenerative Disease, UCL Institute of Neurology, London, UK
2. Department of Genetics, Evolution and Environment, Institute of Healthy Ageing, University College London, London, UK
3. Maurice Wohl Clinical Neuroscience Institute, Department of Basic and Clinical Neuroscience, King’s College London, UK

* These authors contributed equally

Correspondence to:

Ammar Al-Chalabi

Professor of Neurology and Complex Disease Genetics

Maurice Wohl Clinical Neuroscience Institute

Cutcombe Road

King’s College London

London SE5 9RX, UK

[ammar.al-chalabi@kcl.ac.uk](mailto:ammar.al-chalabi@kcl.ac.uk)

**King's ALS Staging SOP**

***This procedure takes the form of a semi-structured interview. It is hierarchical and is not therefore a functional scale. In all cases, if involvement of a region is obvious to an untrained observer it should be counted as involved. Only findings related to ALS should be scored.***

1. Stage 4 has been reached if there is explicit evidence of **feeding failure secondary to ALS** (defined as **weight loss 10% or more** of premorbid weight, or there is a **stated recommendation of gastrostomy** by the multidisciplinary team) or **respiratory failure secondary to ALS** (which **clearly meets the definition** according to the NICE Guidelines, summarized below in notes parts A and B). If gastrostomy or NIV are recommended but the intervention is *refused* by the patient, Stage 4 has still been reached.

2. To assess earlier stages the El Escorial regions are considered. The number of regions involved defines the stage. Abnormal reflexes may suggest involvement of a region (relevant reflexes are detailed below with each region subheading). *Any deep tendon reflexes* may be examined at the time of interview if the interviewer is clinically trained, or they can be taken from the most recent clinical examination. If an appropriate recent clinical examination is not available, the following abnormal signs can be tested by a suitably trained non-clinical examiner: a pathologically brisk jaw jerk, pectoral reflexes, Hoffman’s sign, crossed adductor reflexes, pathologically brisk patellar reflexes or ankle clonus.

*Bulbar:*

Involvement defined as slurred speech, dysphonia, problems swallowing liquids, choking. Examination showing tongue atrophy, fasciculation, slowness of movement or a pathologically brisk jaw jerk reflex is acceptable as an alternative if no symptoms are reported.

*Upper limbs:*

Involvement defined as trouble with keys, doors, buttons, zips or carrying bags, reported by the patient. Examination showing wasting of the first dorsal interosseus, pectoral reflexes or Hoffman’s sign is acceptable as an alternative if no symptoms are reported. (A clinician may examine any reflexes and draw their own conclusion).

*Lower limbs:*

Involvement defined as stiffness, spasm or cramping, falls, or the foot catching on walking. Examination showing gait stiffness or foot drop, crossed adductor reflexes, pathologically brisk patellar reflexes or ankle clonus is acceptable as an alternative if no symptoms are reported. (A clinician may examine any reflexes and draw their own conclusion). Extensor plantar responses are *not* acceptable as suggesting of involvement.

***Notes***

*A. Definition of respiratory failure*

SNP:

In the absence of symptoms, respiratory failure is defined as sniff nasal inspiratory pressure (SNP) <40cm H_2_O or decrease > 10cm H_2_O per 3 months. If there are symptoms of orthopnoea or excessive daytime sleepiness, respiratory failure is defined as SNP <65cm H_2_O for men and 55cm H_2_O for women.

*B. Other acceptable definitions of respiratory failure*

FVC:

In the absence of symptoms, respiratory failure is defined as FVC <50% predicted. If there are symptoms of orthopnoea or excessive daytime sleepiness, respiratory failure is defined as FVC <80% predicted.

Oximetry measures:

Respiratory failure is defined as SpO_2_ at 94% or lower, with either pCO_2_ >6kPa, or overnight oximetry showing >5 dips per hour to SpO_2_ <80%.

Symptoms of potential respiratory impairment include dyspnoea, orthopnoea, morning headaches, fatigue, disturbed sleep, nightmares, poor concentration and/or memory, recurrent chest infections, confusion and hallucinations. Signs of potential respiratory impairment include tachypnoea, shallow breathing, weak cough or sniff, use of accessory muscles and reduced chest expansion.

Derived from the NICE Guideline at <https://www.nice.org.uk/guidance/ng42/resources/motor-neurone-disease-assessment-and-management-pdf-1837449470149>.

*C. Please note the following examples of clinical findings which do not meet staging criteria for involvement:*

1. Extensor plantar reflexes with no other lower limb involvement do not satisfy criteria for involvement of the lower limbs.
2. Dysphagia not secondary to ALS or without weight loss greater than the 10% threshold and respiratory symptoms not fulfilling NICE respiratory failure guidelines do not meet Stage 4 criteria.
3. Fasciculation without wasting, weakness or reflex changes does not constitute involvement of a limb. It is acceptable as indicating involvement in the tongue.

For further information, please also refer to the ENCALS outcome measures website: <https://www.encals.eu/outcome-measures>.

**Case Vignettes**

**Introduction**

This workshop is split into two tasks. In the first task you will be asked to intuitively stage some clinical vignettes from Stage 1 (early stage disease) to Stage 4 (late stage disease). In the second task you will analyse the same vignettes using a clinical staging system that will be taught during the workshop.

**ALS Clinical Staging**

In all these cases the patients described have a diagnosis of amyotrophic lateral sclerosis (ALS), with clinical examination and investigations consistent with this diagnosis.

***Case 1:*** A 59 year old man describes a worsening history of difficulty with keys, turning door handles, and manipulating buttons over the last year. His arm has become noticeably thinner in that time, and occasionally the muscles twitch. He cannot straighten the fingers of his right hand easily. He notices his walking has also slowed down, so that whereas he could previously walk a mile in about 15 minutes, it now takes about 30. There is no problem with speaking or swallowing, and there are no respiratory symptoms. Clinical examination confirms distal weakness of the right upper limb and proximal wasting and weakness of the lower limbs, with brisk reflexes in some muscles.

***Case 2:*** A 65 year old man developed problems with walking two years ago. He initially noticed his right foot was dragging, and occasionally he would trip over. Eighteen months ago he started finding it increasingly difficult to lift heavy objects and to dress himself. Over the past 6 months he has found his speech has become slurred, although he has not had difficulty with chewing or swallowing food. He was previously able to walk for a mile without becoming short of breath, but now after 10 minutes he feels breathless. He also becomes short of breath when lying flat at night and he wakes up with a headache. His clinical examination reveals weakness of both lower limbs, primarily on the right side, and some proximal weakness in his upper limbs, with pathologically brisk reflexes. His peripheral capillary oxygen saturation on room air is 93% and an arterial blood gas test reveals that he is hypercarbic with pCO_2_ of 6.3 kPa.

***Case 3:*** A 60 year old woman reports a two and a half year history of difficultly walking, and she finds that recently this has become worse. Her main problem is that her left leg feels weak, and she finds it drags while she is walking. She also notices twitching in her leg muscles. She now is unable to walk for longer than 10 minutes. A few weeks ago she started having weakness in her right hand, finding that she cannot easily use her right thumb when picking up small objects. She has not experienced any difficulty swallowing or with speech, or with her breathing. Clinical examination reveals she has distal wasting and weakness of both lower limbs and her right hand and a pathologically brisk jaw jerk reflex.

***Case 4:*** A 71 year old man reports a seven month history of pain and weakness in his right leg making walking difficult. He has not noticed any weakness in his arms, or any problems swallowing or with his speech. His breathing has also been normal. Clinical examination confirms he has proximal right lower limb weakness. Several fasciculations are noted in his right upper limb and he has pathologically brisk reflexes in all limbs with bilateral finger jerks and Hoffmann's jerks elicited.

***Case 5:*** A 63 year old man has a fourteen month history of slurred speech, finding this has become worse over the past four months. His speech is now difficult to understand and his voice has become softer, so he has to repeat himself frequently. He notices he is has excessive saliva, and finds it difficult to swallow this, so he frequently drools. Over this same period of time he finds himself choking whilst eating solid food, so he now can only manage a food of a very soft consistency or liquid food, but occasionally chokes on this also. His clinicians recommend that he should now consider having a gastrostomy inserted, but he decides against this intervention.

***Case 6:*** A 75 year old man describes a six month history of difficulty using his right arm. He finds he cannot carry heavy objects and now finds it problematic combing his hair and brushing his teeth with his right arm. He sometimes finds it difficult to cut up food and notices handwriting is slower and clumsier. He has no difficulty with walking or with his speech, swallowing or breathing. Clinical examination reveals proximal wasting and weakness of his right upper limb with pathologically brisk upper limb reflexes.

***Case 7:*** A 41 year old man noticed cramps in his left arm nine months ago with weakness, and now his right arm and hand are also weak. He can no longer work as a builder as he cannot lift his building materials and tools. He sometimes also finds it difficult to tie his shoelaces and fasten zips and buttons. He is still able to walk to the local train station one mile away, and does not feel that his legs are weak, that his walking has slowed down or that he has difficulty climbing stairs. On clinical examination his strength is reduced in the upper limbs and normal in the lower limbs with pathologically brisk knee and ankle reflexes and crossed adductor reflexes. Plantars are downgoing.

***Case 8:*** A 66 year old woman has a four year history of worsening weakness of her left leg, leading to difficulty walking. She now uses a walking stick around the house, and before her symptoms started she could walk a mile, but can now no longer do this. Over the past two years she has also noticed difficulty lifting her grandson and finds it challenging to cut up food and dress herself. Over the past few months she occasionally gets breathless at night and on exertion. She does not sleep well, finding she is sleepy during the day. Her peripheral capillary oxygen saturation on room air is 95% and her other respiratory tests are within normal limits.

***Case 9:*** A 77 year old man has had a two year history of progressive difficulty with walking. He used to be able to walk a mile in 20 minutes, but now cannot walk that far without his legs feeling stiff. He has not had any weakness in his arms, and has not had difficulty swallowing food, with his speech or breathing. His clinical examination confirms proximal weakness, increased tone and pathologically brisk reflexes in his lower limbs, but is otherwise normal. He had a gastrostomy tube inserted four months ago due to oropharyngeal cancer, which is being treated with surgery, chemotherapy and radiotherapy.

***Case 10:*** An 84 year old woman has progressive dysarthria and excessive salivation for seven months. On examination she has a wasted tongue, a pathologically brisk jaw jerk and bilateral wasting of the small muscles of her hands, including the first dorsal interossei. The rest of the examination is normal.

***Case 11:*** A 72 year old woman has a fourteen month history of dragging her right foot while walking. Her left leg also became affected three months ago and she has no other weakness. She is found by her doctor to have pathologically brisk patellar reflexes and brisk biceps and supinator reflexes in her right arm. All other examination findings are normal.

***Case 12:*** A 55 year old woman describes a ten month history of weakness and stiffness in both hands. A few months later she starts to limp with her right foot slapping on the ground. She has no reported problems with her speech or swallowing, and no respiratory symptoms. On examination she has a wasted, fasciculating tongue and distal wasting and weakness in her limbs. During the clinic visit her voice becomes quieter and she slurs her sentences. Respiratory testing shows the vital capacity is 75% of predicted.

***Case 13:*** A 46 year old man has a nine month history of progressive weakness of the legs. On examination he has increased tone in all four limbs with wasting and fasciculation in his lower limbs and crossed adductor reflexes.

***Case 14:*** A 57 year old man noticed muscle wasting in his right hand two years ago. Last year his right foot started to flap when he was walking. Over the past two months his speech has become slurred after talking for a long time. Examination reveals a wasted and fasciculating tongue, weakness of the right hand and a right foot drop.

***Case 15:*** A 70 year old man has a year long history of difficulty swallowing, choking and excessive salivation. He becomes very fatigued when eating and suffers from frequent chest infections despite a modified diet. His weight has dropped from 65 Kg to 55 Kg.

***Case 16:*** A 54 year old woman reports weakness in her left leg slowly getting worse over the last three years. Over the past year her arms have become weaker. For the past few months she has been feeling short of breath and sleeping poorly. Her forced vital capacity (FVC) is less than 50% of the predicted value.

***Case 17:*** A 70 year old woman reports a four year history of difficulty walking requiring walking aids with no other symptoms. Examination reveals increased tone in the all the limbs, more so on the right side. She has a pyramidal pattern of weakness in all limbs, and is unable to lift her arms. She has pathologically brisk reflexes in her limbs. There are no bulbar signs, no weight loss, and respiratory testing is normal.

**Answers:**

| **Case Number** | **King’s ALS stage** |
| --- | --- |
| 1 | 2 |
| 2 | 4 |
| 3 | 3 |
| 4 | 2 |
| 5 | 4 |
| 6 | 1 |
| 7 | 2 |
| 8 | 2 |
| 9 | 1 |
| 10 | 2 |
| 11 | 2 |
| 12 | 3 |
| 13 | 2 |
| 14 | 3 |
| 15 | 4 |
| 16 | 4 |
| 17 | 2 |
